# RNA-binding proteins as a point of convergence of the PI3K and p38 MAPK pathways

**DOI:** 10.3389/fimmu.2012.00398

**Published:** 2012-12-26

**Authors:** Ram K. C. Venigalla, Martin Turner

**Affiliations:** Laboratory of Lymphocyte Signalling and Development, The Babraham InstituteBabraham, UK

**Keywords:** RNA-binding proteins, PI3K, MAPK

## Abstract

Understanding the mechanisms by which signal transduction pathways mediate changes in RNA abundance requires the examination of the fate of RNA from its transcription to its degradation. Evidence suggests that RNA abundance is partly regulated by post-transcriptional mechanisms affecting RNA decay and this in turn is modulated by some of the same signaling pathways that control transcription. Furthermore, the translation of mRNA is a key regulatory step that is influenced by signal transduction. These processes are regulated, in part, by RNA-binding proteins (RBPs) which bind to sequence-specific RNA elements. The function of RBPs is controlled and co-ordinated by phosphorylation. Based on the current literature we hypothesize that RBPs may be a point of convergence for the activity of different kinases such as phosphoinositide-3-kinase and mitogen-activated protein kinase which regulate RBP localization and function.

## INTRODUCTION

The response of cells to environmental stimuli frequently involves changes in gene expression. This may be controlled at multiple levels including the production of new RNA by transcription. Post-transcriptional regulation at the RNA level includes nuclear RNA processing (frequently a co-transcriptional process), as well as RNA export, decay, localization, and translation. These processes are integrated with changes in protein stability and function. Signaling pathways are a major mechanism for co-ordination of these distinct mechanisms (Schoenberg and Maquat, 2012).

Substantial evidence obtained over two decades has highlighted the importance of mRNA stability in gene regulation (Cheadle et al., 2005; Keene, 2007; Anderson, 2008; Hao and Baltimore, 2009). The half-life of different mRNAs can vary from 15 min to more than 24 h depending on the activation status of a cell, for example, the half-life for interleukin-2 (IL-2) mRNA is 17 min in non-stimulated T cells but upon activation with anti-CD3/CD28 the half-life for IL-2 mRNA is increased to 232 min (Raghavan et al., 2002, 2004; Yang et al., 2003). The mRNA half-lives in bacteria (Bernstein et al., 2002) and yeast (Wang et al., 2002) are mostly shorter in comparison to mammalian cells. The increased mRNA half-life correlates with increasing organismal complexity and a tendency for 3′ untranslated regions (UTR) within mRNA to become longer in more complex species (Mazumder et al., 2003; Dinger et al., 2011). Thus, post-transcriptional regulation of mRNA may be a more prevalent amongst complex multicellular organisms.

The difference in mRNA half-lives can lead to significant changes in the abundance of mRNA (Ross, 1995). This was illustrated in genome-wide studies which have shown that up to 50% of altered mRNA abundance in lymphocytes is due to the regulation of mRNA stability (Lam et al., 2001; Cheadle et al., 2005). The stability of mRNA is regulated by distinct sequences present in the coding and UTR of mRNA (Caput et al., 1986; Shaw and Kamen, 1986; Schoenberg and Maquat, 2012). Conservation of these sequences within the UTR region among different species further emphasizes their regulatory role.

The fate of RNA can be regulated by the interplay between sequences within the RNA (*cis*-acting) and *trans*-acting factors present in the nucleus and cytoplasm (Keene, 2007; Anderson, 2010; Elkon et al., 2010). *Trans*-acting factors such as non-coding RNA (Rinn and Chang, 2012), microRNA (Fabian and Sonenberg, 2012), and RNA-binding proteins (RBPs) have been reported to regulate mRNA stability and translation. Translation can also be regulated through control of the length of the poly(A) tail in the cytoplasm (Weill et al., 2012). The class of *trans*-acting factors we will focus on in this review are the RBPs. The function of RBPs can be controlled by different signaling pathways and several excellent reviews covering the detailed regulation of RBPs in response to stress pathways have been published (Eberhardt et al., 2007; Doller et al., 2008; Sandler and Stoecklin, 2008; Kim et al., 2010). Here we will discuss the function of selected RBPs at the molecular level and how they are being controlled by phosphoinositide-3-kinase (PI3K) and mitogen-activated protein kinases (MAPK) signaling.

## RNA-BINDING PROTEINS

**FIGURE 1:**
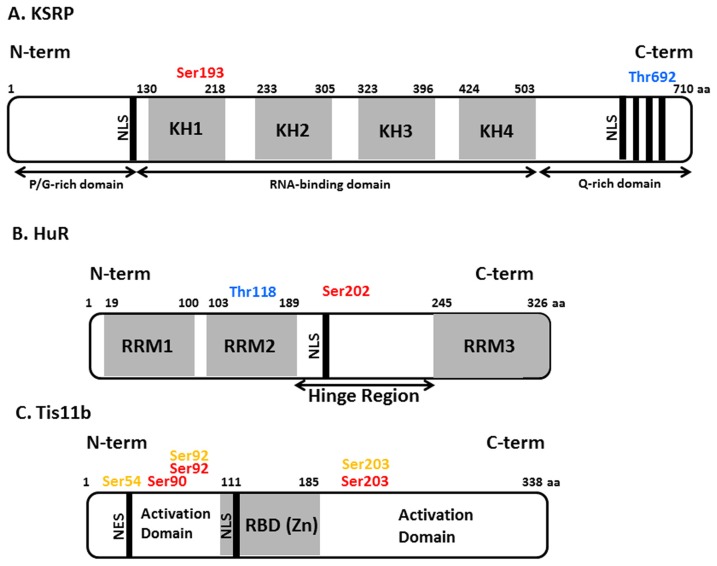
**Schematic view of domains and phosphorylation sites in KSRP, HuR and Tis11b proteins**. **(A)** KH domains and nuclear localization signals are represented in light gray and black colors respectively. The amino acid numbers are depicted on top of protein domain structure. Proline/glycine-rich and glutamine-rich domains on N-terminal and C-teriminal end respectively are marked. **(B)** Hinge region (containing nucleocytoplasmic sequence and nuclear localization signal, NLS); RNA recognition motifs (RRM) and their corresponding amino acid positions are depicted. **(C)** Activation domains on N- and C-terminal ends, NLS, nuclear export sequence (NES), and RBD (RNA-binding domain) are shown. The experimentally determined phosphorylation sites on RBPs are given colors in following manner, red: PKB phosphorylation sites, blue: p38 phosphorylation sites, yellow: MK2 phosphorylation sites.

It has been estimated that approximately 1000 RBPs are encoded in the mammalian genome (Keene, 2007; Araujo et al., 2012; Baltz et al., 2012; Castello et al., 2012). These play important roles in splicing, nuclear export, mRNA stability, localization, and translation. RBPs exert their function by physically interacting with RNA and can do so in a sequence-specific manner. Amongst the well-characterized sequences that bind RBPs are the adenine- and uridine-rich elements (ARE). RBPs that bind to ARE include KSRP as well as TTP (TIS11) and its homologs TIS11b (also called BRF-1; butyrate response factor-1), and TIS11d (BRF-2). These have been shown to promote ARE-dependent mRNA decay but may also affect translation. HuR and its close relatives have also been suggested to promote mRNA stability and to regulate translation (Srikantan and Gorospe, 2012; Yiakouvaki et al., 2012). The domain structures of these RBPs are depicted in **Figure 1**.

## CONTROL OF RBP FUNCTION BY SIGNAL TRANSDUCTION PATHWAYS

Diverse stimuli (such as chemical and environmental mediators, reviewed in Eberhardt et al., 2007) induce signaling cascades which control RBP function by means of protein phosphorylation. However the details of how these signaling pathways control the abundance and RNA-binding properties of RBPs at molecular level are only beginning to be understood. Amongst the signaling kinases discussed below and summarized in **Table 1** are PI3K, PKB (protein kinase B), mTOR (mammalian target of rapamycin), and p38 MAPK. Experimentally determined phosphorylation sites on the RBPs we will focus on are depicted in **Figure 1**. A more extensive list derived from phospho-proteomics studies is given in **Table 2**.

**Table 1:** Representative list of signaling kinases regulating RBP function.

RBP	Kinase	Phosphosite/ inhibitor/siRNA	Type of cell	Target gene	Fate of RBP or its target gene function	Reference
TIS11	Protor-2	siRNA	Jurkat	TNF-α, COX-2, GM-CSF, IL-3	Regulate RBP function	Holmes et al. (2012)
TIS11	MK2	siRNA	HPMECs	ICAM-1 and IL-8	Target gene mRNA stability	Shi et al. (2012)
TIS11	MK2	Ser52, Ser178	3T3		Inhibition of mRNA decay property, RBP stability	Chrestensen et al. (2004)
TIS11b	PKB	Ser92, Ser203	HT1080 and MEF		Inhibition of mRNA decay property, RBP stability	Schmidlin et al. (2004)
TIS11b	MK2	Ser54, Ser92, Ser203	HT1080	GM-CSF	Inhibition of mRNA decay property	Maitra et al. (2008)
HuR	mTOR	siRNA	RIE-1	Ornithine decarboxylase	Target gene mRNA stability	Origanti et al. (2012)
HuR	MK2	Inhibitor	184B5/HER	COX-2	Target gene mRNA stability	Subbaramaiah et al. (2003)
TIA1	mTOR	Inhibitor	HeLa	5′ terminal oligopyrimidine tracts	Translation repressor	Damgaard and Lykke-Andersen (2011)
TIAR	mTOR	Inhibitor	HeLa	5′ terminal oligopyrimidine tracts (5′TOP)	Translation repressor	Damgaard and Lykke-Andersen (2011)
KSRP	PKB	Ser193	Alpha-T3-1	β-catenin	Inhibition of mRNA decay property	Gherzi et al. (2006)
NF90	PKB	Ser647	Jurkat	IL-2	Stabilizing mRNA	Pei et al. (2008)
hnRNP F	mTOR	Inhibitor	HEK293	controls proliferation		Goh et al. (2010)
CELF1	PKB	Ser28	Myoblasts	CcnD1		Salisbury et al. (2008)
Nucleolin	PI3K	Inhibitor	Endothelial cell line	KLF2	Up regulation of KLF2	Huddleson et al. (2006)
IMP2	mTOR	Ser162/164	Human RD	IGF2	Regulate translation of IGF2	Dai et al. (2011)
YB1	PKB	Ser99	CEF		Regulate translation capacity of YB1	Bader and Vogt (2008)

*Table 1 shows type of kinase and/or its phosphorylation site on RBP function over its target gene in a specific cell. In studies where phosphorylation site is not reported, authors used either inhibitor or siRNA against studied kinase to analyse its role on RBP function.*

**Table 2:** Phosphorylation sites on RBP from phospho-proteomic studies.

RBP	Kinase	Phosphorylation site	Type of cell	Reference
TIS11	MK2	Ser52	3T3	Wang et al. (2008)
TIS11	CAMK2	Ser52	CD8 T	Navarro et al. (2011)
TIS11	PKB	Ser248	CD8 T	Navarro et al. (2011)
TIS11	PKA	Ser197	HEK-293	Cao et al. (2006)
TIS11	GSK3	Ser218	HEK-293	Cao et al. (2006)
TIS11	ERK	Ser228	HEK-293	Cao et al. (2006)
TIS11b	PKA/PKB	Ser54/92	CD8 T	Hsu et al. (2011); Navarro et al. (2011)
TIS11b	mTOR	Ser334	MEF	Hsu et al. (2011)
TIS11d	PKB	Ser98	3T3	Wang et al. (2008)
TIS11d	PKA/PKB	Ser28/98	CD8 T	Navarro et al. (2011)
TIS11d	mTOR	Ser57/73/416/464	MEF	Hsu et al. (2011)
KSRP	mTOR	Ser182, Ser185	MEF	Yu et al. (2011)
KSRP	PKA	Ser481	CD8 T	Navarro et al. (2011)
Nucleolin	mTOR	Ser28/34/40/41/145/157/616/ 189/403/212/460,Thr121	MEF	Yu et al. (2011)
Roquin	ERK/MAPK	Ser770	CD8 T	Navarro et al. (2011)
Roquin	mTOR	Ser531/535	MEF	Hsu et al. (2011)
hnRNPk	CDK2	Ser284	CD8 T	Navarro et al. (2011)
hnRNPu	CK1	Ser187	CD8 T	Navarro et al. (2011)
hnRNPu	CAMK2	Ser247	CD8 T	Navarro et al. (2011)
hnRNPF	mTOR	Ser63	MEF	Hsu et al. (2011)
hnRNPA3	mTOR	Ser356/359/367	MEF	Hsu et al. (2011)
hnRNPA3	CK1	Ser359	CD8 T	Navarro et al. (2011)
hnRNPA3	PKA	Ser357	CD8 T	Navarro et al. (2011)
hnRNPAb	PKA	Ser260	CD8 T	Navarro et al. (2011)
AUF1	mTOR	Ser82/83, Thr177	MEF	Hsu et al. (2011)
hnRNPA2B1	mTOR	Ser245/247/266/272, Tyr254	MEF	Hsu et al. (2011)
hnRNPK	mTOR	Ser284/379	MEF	Hsu et al. (2011)
hnRNPC	mTOR	Ser229/232/241/268/306/313	MEF	Hsu et al. (2011)
hnRNPUL1	mTOR	Ser513	MEF	Hsu et al. (2011)
hnRNPUL1	CAMK2	Ser195	CD8 T	Navarro et al. (2011)
hnRNPA1	mTOR	Ser6/257	MEF	Hsu et al. (2011)
hnRNPA1	CDK1	Ser6	CD8 T	Navarro et al. (2011)
hnRNPH2	mTOR	Ser104	MEF	Hsu et al. (2011)
HuR	mTOR	Ser202	MEF	Yu et al. (2011)
IMP2	mTOR	Ser102	MEF	Yu et al. (2011)
IMP2	mTOR	Ser160/161/163	MEF	Hsu et al. (2011)

*The phosphorylation sites on RBPs for respective kinases in a specific cell are obtained from published phospho-proteomic studies. In Yu et al. (2011)), mTOR phosphorylation sites on RBP in MEFs are derived using inhibitors rapamycin and KU-0063794 against mTOR in a SILAC experiment. Similarly in Hsu et al. (2011), mTOR regulated phosphosites on RBPs in MEFs were verified using Torin1 (mTOR) inhibitor in an iTRAQ experiment. By employing SILAC technology, Navarro et al. (2011) identified phosphorylation sites on different RBPs for the respective kinases in TCR stimulated CD8 T cells. Wang et al. (2008) identified phosphorylation sites on RBPs in 3T3 cells using iTRAQ.*

### THE PI3K PATHWAY

The PI3K pathway plays an important role in controlling cell growth, differentiation, survival, chemotaxis, and metabolism. The activation of PI3K stimulates the generation of phosphatidylinositol 3,4,5 trisphosphate at the cell membrane, which mediates the recruitment of PKB (also known as AKT) and phosphoinositide-dependent kinase 1 (PDK1). PKB is then activated following phosphorylation by PDK1 at Thr^308^. Mammalian target of rapamycin complex 2 (mTORC2) phosphorylates PKB at hydrophobic motif (Ser^473^; Jacinto et al., 2006; Sarbassov et al., 2006; Facchinetti et al., 2008). PKB, which has three isoforms encoded by different genes, is involved in regulating cell proliferation and survival (Pearce et al., 2010). Genetic alterations in the PI3K pathway, such as mutations in catalytic subunits and loss of the negative regulator PTEN have been found in cancers (Kok et al., 2009). PI3K also regulates immunity and inflammation by controlling the recruitment and activation of immune cells. Thus, PI3K is an important signal integrator in maintaining immune homeostasis.

An early report implicated PI3K in the regulation of the stability of IL-3 mRNA by TIS11/TTP but did not identify phosphorylation sites within TTP (Ming et al., 2001). Subsequent studies using mass spectroscopy identified over 30 phosphorylation sites on TTP (Cao et al., 2006). Both Ser^60^ and Ser^113^ in human TTP are predicted to be PKB phosphorylation sites (Cao et al., 2007) but this has not been experimentally confirmed.

Following activation of the PI3K pathway the phosphorylation of TIS11b by PKB at Ser^90^, Ser^92^, and Ser^203^ facilitates its binding with 14-3-3. This sequesters TIS11b in the cytoplasm and inhibits its ability to promote mRNA decay (Schmidlin et al., 2004; Benjamin et al., 2006). Phosphorylation of these sites appears to increase the stability of the TIS11b protein which is consistent with a shorter half-life/increased degradation of TIS11b protein in PKBα knockout mouse embryo-derived fibroblasts (MEFs; Benjamin et al., 2006). Mutagenesis of Ser^90^, Ser^92^, and Ser^203^ in TIS11b uncoupled it from regulation by PKB and the mutated protein, which retained the ability to promote RNA decay, could no longer associate with 14-3-3 (Benjamin et al., 2006).

KSRP mediates mRNA destabilization by binding to AREs in target mRNAs. Phosphorylation of KSRP at Ser^193^ by PKB facilitates its binding to 14-3-3 which inhibits its interaction with the RNA decay machinery (in this case the exosome; Gherzi et al., 2006). In this way, KSRP is prevented from promoting the degradation of β-catenin mRNA (Gherzi et al., 2006). Phosphorylation at Ser^193^ creates a binding site for 14-3-3ζ in the N-terminal KH domain of KSRP (Diaz-Moreno et al., 2009) which, upon interaction with KSRP, promotes its nuclear localization. Thus, the availability of KSRP in cytoplasm and its ability to mediate mRNA decay is limited by phosphorylation. Interestingly, phosphorylation at Ser^193^ redirects the function of KSRP to become a regulator of the maturation of miRNA (Trabucchi et al., 2009). In C2C12 myoblasts PI3K-dependent phosphorylation of KSRP enhanced its ability to accelerate myogenic miRNA processing while attenuating its ability to promote myogenic mRNA decay. Thus, during myogenesis, KSRP appears to function as a dynamic switch controlling RNA regulated by PI3K (Briata et al., 2012).

The importance of the PI3K pathway in controlling mRNA decay was further illustrated by a recent study which demonstrated that approximately 20 out of 50 transcripts regulated by PI3K were affected at the level of mRNA stability (Graham et al., 2010). Using siRNA knockdown experiments TIS11b and KSRP were shown to be involved in stabilization of the mRNAs of down-regulated genes (Graham et al., 2010). Interestingly Graham et al., 2010) observed no effect on mRNA stability by RBPs such as AUF1 and HuR which are not known to be controlled by PI3K signaling, suggesting a key role of PI3K pathway in maintaining mRNA stability via TIS11b and KSRP.

## MAMMALIAN TARGET OF RAPAMYCIN

The mTOR is a kinase component of signaling complexes which play very important roles in immune cell function (Powell et al., 2012). The mTORC1 complex can be activated in a PI3K-dependent or independent manner and is highly susceptible to inhibition by rapamycin. By contrast, mTORC2 which phosphorylates PKB Ser^473^ and contributes to its activation is much less sensitive to rapamycin (Jacinto et al., 2006; Sarbassov et al., 2006; Facchinetti et al., 2008). mTOR can also be activated in a PI3K-independent manner by spleen tyrosine kinase (SYK), as reported in follicular lymphoma cells (Leseux et al., 2006). In addition to SYK, the Erk pathway can also activate mTOR (Shaw and Cantley, 2006). Thus, mTOR activated in a PI3K-dependent or independent manner exerts effector functions via a number of targets including PKB (Powell et al., 2012).

In a phospho-proteomic study the Blenis Group reported Ser^182^ and Ser^185^ as phosphorylation sites on KSRP for mTOR. These findings were verified by treating MEFs with the mTOR inhibitors rapamycin and KU-0063794 (**Table 2**; Yu et al., 2011). The same phosphorylation sites in KSRP were predicted by PHOSIDA (http://www.phosida.com; the post-translational modification database which provides information on the sites of phosphorylation, *N*-glycosylation, and acetylation across nine different species; Gnad et al., 2007; Gnad et al., 2011). However Ser^185^ of KSRP has also been predicted to be a casein kinase 1 (CK1) phosphorylation site and the function of this phosphorylation is presently unknown.

The Blenis group also reported HuR Ser^202^ as a phosphorylation site for mTOR and this finding was substantiated using the mTOR inhibitors rapamycin and KU-0063794 in MEFs (**Table 2**; Yu et al., 2011). However, limited evidence exists for the functional regulation of HuR by mTOR as it has not yet been reported whether or not mTOR regulates HuR localization and function. Previously the Gorospe group had reported Ser^202^ as a target for cyclin-dependent kinase 1 (Cdk1; Kim et al., 2008). Phosphorylation of HuR by Cdk1 promoted its movement into the nucleus where HuR appeared to be associated with 14-3-3 proteins. A modified HuR protein with a non-phosphorylatable serine to alanine mutation resided predominantly in the cytoplasm. Unphosphorylated HuR bound poorly to 14-3-3, which increased the availability of HuR for stabilizing its target mRNAs (Kim et al., 2008). Thus, the function of HuR is modulated by Cdk1 during the cell cycle (Blethrow et al., 2008; Kim et al., 2008). In another very recent report CDK5 phosphorylation of HuR at Ser202 has been shown to regulate its function in cell cycle progression (Filippova et al., 2012). HuR regulates ornithine decarboxylase (ODC) mRNA stability (Nowotarski and Shantz, 2010) and the binding of HuR to the ODC transcript is decreased when mTORC1 signaling is inhibited using rapamycin, an mTORC1 inhibitor (Origanti et al., 2012). The mTOR mediated phosphorylation at Ser^202^ on HuR might be one mechanism through which mTOR regulates proliferation.

Protor2, a component of mTORC2 kinase has been shown to bind to TTP in Jurkat cells following treatment with carbonyl cyanide 4-(trifluoromethoxy) phenylhydrazone, an uncoupler of mitochondrial oxidative phosphorylation. The interaction between TTP and Protor2 was suggested to be necessary for enhancing TTP-mediated turnover of mRNAs such as IL-3, GM-CSF, COX-2, and TNF (Holmes et al., 2012). The siRNA knockdown of protor2 inhibited the localization of TTP to mRNA processing bodies (P-bodies), the sites where mRNA decay enzymes are concentrated (Parker and Sheth, 2007). Very recently mTOR has been reported to regulate iron homeostasis by modulating transferrin receptor 1 (TfR1) stability via TTP (Bayeva et al., 2012).

## THE P38 MAPK PATHWAY

Mitogen-activated protein kinases are major regulatory hubs where inflammation and stress responses are regulated. Three major MAPK pathways are p38, JNK, and ERK. We discuss below the findings implicating p38 and its substrate MAPK activated protein kinase 2 (MK2) in regulating RBP activity.

The p38 pathway via MK2 regulates the mRNA decay property and the mRNA and protein expression of TTP (Dean et al., 2001; Tchen et al., 2004; Brook et al., 2006; Hitti et al., 2006). MK2 phosphorylates mouse TTP at Ser^52^ and Ser^178^ which stabilizes TTP protein (Brook et al., 2006). Upon dephosphorylation of these sites, TTP moves from the cytoplasm to the nucleus and undergoes degradation (Brook et al., 2006). MK2-mediated phosphorylation of TTP does not affect its binding to target mRNA, but inhibits the ability of TTP to recruit deadenylases to target mRNA for their degradation in cytoplasm (Carballo et al., 2001; Stoecklin et al., 2004; Clement et al., 2011). Further investigation of this demonstrated that carbon catabolite repressor protein 4-associated factor-1 (CAF1) was the major source of deadenylase activity responsible for TTP-directed deadenylation (Marchese et al., 2010). MK2 phosphorylation reduced the ability of TTP to promote deadenylation by inhibiting the recruitment of CAF1 deadenylase independently of 14-3-3. The Stoecklin group demonstrated that Not1, a component of carbon catabolite repressor protein 4 (Ccr4)-negative on TATA (NOT) complex, associates with TTP and is required for the decay of ARE-mRNAs (Sandler et al., 2011). Subsequently it has been reported that TTP regulates the translation of TNF mRNA at the endoplasmic reticulum (Tiedje et al., 2012). Phosphorylation of TTP by MK2 weakens its ability to bind to TNF mRNA and allowed HuR-binding to TNF mRNA which promoted its translation. Conflicting data exists on the ability of phospho-TTP to bind its target mRNA (Clement et al., 2011; Tiedje et al., 2012) The former group found that phosphorylation did not alter TTP binding to its target mRNA but the latter group found that phosphorylation reduced the affinity of TTP binding to its target mRNA.

TIS11b is also regulated by MK2. The phosphorylation of TIS11b at Ser^54^, Ser^92^, and Ser^203^ by MK2 inhibits the ability of TIS11b to promote ARE-mediated mRNA decay (Maitra et al., 2008). The phosphorylation-dependent inhibitory effects of MK2 on TIS11b do not seem to alter its ability to bind RNA or its association with mRNA decay enzymes. Furthermore, the MK2-mediated effects on TIS11b were independent of PKB (Maitra et al., 2008).

Several studies indicate that the function of HuR is regulated by the p38 pathway. The abundance of COX-2 mRNA is controlled by the p38-dependent regulation of the binding of HuR to the COX-2 3′ UTR (Subbaramaiah et al., 2003). In neuronal cell line p38 activation following treatment with anisomycin promotes the cytoplasmic accumulation of HuR where it interacts with and stabilizes the survival motor neuron (SMN) transcript (Farooq et al., 2009). In mouse splenic T cells, LFA-1 engagement activates p38 which promotes HuR translocation and stabilization of IFN-γ and TNF mRNA (Ramgolam et al., 2010). In none of these studies was it established whether HuR was directly phosphorylated by p38 or MK2. However, an independent study has reported that phosphorylation of HuR at Thr^118^ by p38 promotes its localization to the cytoplasm where it stabilizes p21 mRNA during the DNA damage response (Lafarga et al., 2009). HuR was found to be phosphorylated at Thr^118^ a site previously identified to be phosphorylated by Chk2 (Abdelmohsen et al., 2007). HuR regulated translation of TNF mRNA at the endoplasmic reticulum appeared to be mediated by the effects of the p38 pathway on TTP (Tiedje et al., 2012) and no evidence was found for p38-mediated phosphorylation of HuR. It was suggested that Thr^118^ was instead phosphorylated by Chk2 as a consequence of the over-expression system being used (Tiedje et al., 2012).

The p38 pathway also regulates KSRP. During C2C12 muscle cell differentiation the stability of p21, myogenin, and MyoD mRNA is regulated by p38-mediated phosphorylation of KSRP (Briata et al., 2005). p38 phosphorylates KSRP at Thr^692^ which renders KSRP unable to bind to ARE-containing transcripts thus promoting their stabilization. However, this phosphorylation event does not alter the ability of KSRP to interact with the mRNA degradation machinery (Briata et al., 2005). To our knowledge it has not yet been reported whether or not p38 regulates KSRP function in microRNA maturation.

## ARE RBPs A POINT OF CONVERGENCE FOR PI3K AND P38 SIGNALING?

In NIH 3T3 fibroblasts, the stability of TPA induced IL-3 mRNA is regulated by the p38 and PI3K pathways (Ming et al., 2001). The latter mediates its effects independently of p38 suggesting that p38 and PI3K pathways control IL-3 mRNA turnover by parallel mechanisms. Stabilization of IL-3 mRNA mediated by either of these two pathways is antagonized by TTP and this effect can be overcome by HuR when it is in collaboration with p38 but not with PI3K (Ming et al., 2001). This suggests that signaling pathways activated upon stimulation lead either to activation of stabilizing RBP (HuR) or inactivation of destabilizing RBP (TTP) thus preventing the degradation of transcripts (Ming et al., 2001). Furthermore, another study reported that in U87 glioblastoma cells, the regulation of cyclin D1 and c-Myc mRNA stability by TTP is controlled by p38 in a PKB-dependent manner (Marderosian et al., 2006), implying interdependent roles for p38 and PKB. Data from phospho-proteomic (Cao et al., 2006, 2007; Navarro et al., 2011) and *in vitro* (Chrestensen et al., 2004) studies suggest that MK2 and PKB phosphorylate TTP at Ser^52/178^ and Ser^24^^8^ respectively (**Table 2**).

Both PKB and MK2 target the same phosphorylation sites on TIS11b (Ser^92^ and Ser^203^) and inhibit its mRNA decay activity (Benjamin et al., 2006; Maitra et al., 2008). The mRNA decay property of KSRP is also regulated by PKB and p38 (Briata et al., 2005; Gherzi et al., 2006), but in this instance the kinases do not use the same phosphorylation sites.

**FIGURE 2:**
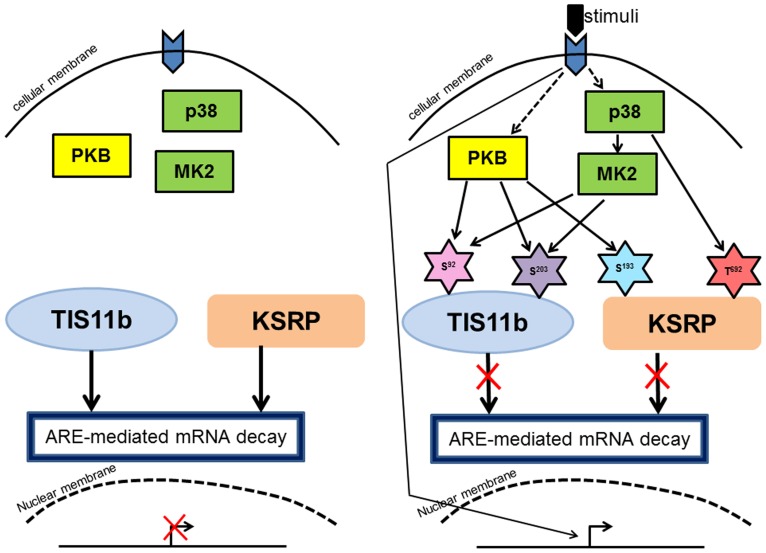
**Convergence of signaling on RBP and its function**. **(A)** In non-activated cells, RBPs (TIS11b and KSRP) are active in promoting mRNA decay of ARE-containing transcripts. **(B)** Upon activation, PKB and MK2 phosphorylate TIS11b at Ser^92^ and Ser^203^ and subsequently TIS11b is prevented in mediating ARE-mediated mRNA decay. Similarly, but not at same phosphorylation sites, KSRP is phosphorylated at Ser^193^ and Thr^692^ by PKB and p38 respectively. These phosphorylation events inhibit KSRP in mediating ARE-mediated mRNA decay. Transcription factors upon activation by signals from kinases drive transcription which is illustrated in the **Figure 2**. The phosphorylation sites are shown in star shape cartoons.

HuR provides a further example of a point of convergence. In this case the circumstances under which the mTOR would affect HuR have not been studied but the phosphorylation site (Ser^202^) has been shown to regulate the function of the protein. Similarly, p38-mediated phosphorylation of HuR at Thr^118^ targets a site previously shown to be targeted by Chk2 (Abdelmohsen et al., 2007). It is clear that multiple different kinases converge on HuR to regulate its function (**Figure 2**).

## FEEDBACK BETWEEN RBPs AND PI3K mTOR PATHWAY

Current literature suggests that kinases control the function of RBPs, however RBPs have also been reported to regulate the expression of kinases suggesting a role for RBPs in feedback control over kinase expression. For example, the 68 kDa Src substrate associated during mitosis (Sam68) is an RBP reported to regulate alternative splicing of mTOR (Huot et al., 2012). In Sam68 knockout cells, intron five is retained in the mTOR transcript introducing a premature termination codon, which results in an unstable mRNA and subsequently reduced protein levels of mTOR. Consequently, the effector pathways of mTOR responsible for adipogenesis are deregulated in these mice, leading to a lean phenotype (Huot et al., 2012). Sam68 is also reported to associate with PI3K in insulin receptor signaling (Sanchez-Margalet and Najib, 2001) and this association may regulate the RNA-binding function of Sam68 (Taylor et al., 1995). Based on this information it is possible that PI3K is regulating RBP function via its downstream kinases and regulating the abundance of its downstream kinases (mTOR) via possible activation of Sam68. Conserved ARE elements in 3′ UTR of p38α, PKBα, PKBγ but not p38β, p38γ, and PKBβ, suggest that these proteins might be targets for regulation of ARE-mediated mRNA stability by RBPs (Gruber et al., 2011).

## CONCLUSION

Gene expression is controlled at the post-transcriptional level by RBPs. However, regulation of the effector function of RBPs on RNA decay and translation is controlled by signals from protein kinases. These effects include inhibition of function by uncoupling from the RNA decay machinery and in some instances reassignment of function. Kinase-dependent relocation of RBP into different compartments of the cell seems to be a common theme amongst structurally diverse RBPs. RBPs represent a class of proteins upon which signaling by the PI3K and p38 pathways converge. Existing literature on this might indicate a potential redundancy of kinases phosphorylating the same serine or threonine amino acid in different cellular functions. A fuller understanding of the interplay between kinases, RBPs and target RNAs may provide important new insights into the dynamic regulation of gene expression.

## Conflict of Interest Statement

The authors declare that the research was conducted in the absence of any commercial or financial relationships that could be construed as a potential conflict of interest.
